# Supplementary material for: Trends of the Florida manatee (Trichechus manatus latirostris) rehabilitation admissions 1991-2017
Source: PLoS One. 2020 Jul 9;15(7):e0223207. doi: 10.1371/journal.pone.0223207 (PMC7347118; doi:10.1371/journal.pone.0223207)
Supplement: Attachment — Submitted filename: PONE-D-19-25939_R1_comments.pdf [file pone.0223207.s003.pdf]

PLOS ONE

Trends of the Florida manatee (Trichechus manatus latirostris) rehabilitation admissions 1991-2017.

--Manuscript Draft--

**Manuscript Number:**	PONE-D-19-25939R1
**Article Type:**	Research Article
**Full Title:**	Trends of the Florida manatee (Trichechus manatus latirostris) rehabilitation admissions 1991-2017.
**Short Title:**	Florida manatee (Trichechus manatus latir[illegible]s) rehabilitation epidemiology
**Corresponding Author:**	Ray Ball, D.V.M.<br>Eckerd College<br>St. Petersburg, FL 33711, FL UNITED STATES
**Keywords:**	manatee; Sirenia; epidemiology; rehabilitation
**Abstract:**	A retrospective study of admission data of 401 West Indian manatees (Trichechus manatus latirostris) presented to the David A. Straz Jr. Manatee Critical Care Center at ZooTampa at Lowry Park (ZooTampa) for rehabilitation from August 1991 through October 2017. Causes of admittance, location of rescue, gender, and age class were all recorded for each manatee admitted. Admittance categories as defined by the Florida Fish and Wildlife Conservation Commission (FWC) included watercraft collisions, natural causes, entanglement, entrapment, orphaned calves, captive born, mothers of rescued calves, calves of rescued mothers, human, and other. The admitted population was primarily from the southwest and northwest coasts and related waterways of Florida. The gender difference was relatively equivocal (54% female) while the adults comprised 79% of the admissions. The overall total admissions increased steadily over the study period as did the admissions for each individual categories of admission. Watercraft collisions and natural causes combined were 71% of all admissions for the entire study period and are the dominant causes of admission. Watercraft collisions are more likely to occur during May through October, whereas natural causes of admittance are more likely to occur between December and March. Rehabilitated manatees may reduce overall manatee mortality and can provide insight into population-based health concerns if evaluated appropriately. Future efforts can incorporate physical examination findings, hematology, biochemistry profiles, and ancillary diagnostic testing to continue to improve the individual welfare of this marine mammal in its natural range. Admissions data could also potentially serve the wider conservation and recovery efforts if it is proven that the data obtained is at least as informative as that obtained by the carcass salvage program. Limited conservation resources could then be re-directed as new challenges arise with the expanding population and potentially expanding range of this species.
**Order of Authors:**	Ray Ball, D.V.M.
	Janice Zgibor
	Marku Malmi
**Response to Reviewers:**	Responses to reviewers for Trends of the Florida manatee (Trichechus manatus latirostris) rehabilitation admissions 1991-2017 by Ball, Malmi, and Zgibor.<br><br>We would like to thank all the reviewers for their efforts in improving our contribution. We have attempted to address all the issues but do have some differences in opinion in what message we are looking to share at this point. These will be detailed below. The first point is that the statistical analysis has been conducted from an epidemiological perspective by epidemiologist in human public health and we believe they are rigorous and yet descriptive enough to be read and understood by readers of various academic backgrounds. With all due respect, we have elected to not alter these methods for this publication.<br>As for the funding statement, we were simply trying to acknowledge supporters of the entire rehabilitation program but no specific funding was obtained in the review and preparation of this manuscript. "The funders had no role in study design, data

Powered by Editorial Manager® and ProduXion Manager® from Aries Systems Corporation

	collection and analysis, decision to publish, or preparation of the manuscript."<br>The causes of admission and death of manatees are defined, as now noted in the manuscript, by the Florida Fish and Wildlife Conservation Commission. In our opinion this reads well and is the vernacular that is used both within our community and with the public at large when communicating about manatees. We are not sure a table would be useful.<br>A detailed map showing all the rescue points would indeed be interesting but we believe too detailed for the purposes intended here. Work of this nature would be most appropriate as part of a thesis. The current manuscript is not delving into outcomes of the manatee rehabilitation; that is a follow-up manuscript so we think the descriptors are adequate. It is indeed an important aspect of the entire endeavor but again we are looking to focus our current efforts to the admission and see if there are lessons we can share.<br>Figures 5 and 6 do look similar but the point of each is distinctly different. Figure 5, with a revised caption, highlights the overall increase in manatee admissions over time in 5 year blocks. Figure 6 highlights the seasonality of admissions based on the admission categories.<br>We agree that some comparison to manatee rescues outside the USA would be appropriate. Brazil is really the only other country that does this with any frequency and that is the basis of the comparison I have added. It becomes problematic to compare facilities with the USA and within Florida as each facility is a private business, both non-profit and profit making operations. Sharing of medical data can be a challenge on an open basis that would include publication. One objective of this manuscript is to start to break down that barrier. With the completion of this manuscript, perhaps other facilities will then be open to compile their own information and data can be compared then. The fact that such a review as we are undertaking has not taken place yet really speaks volumes. This point is actually mentioned in the manuscript in lines 212 - 214.<br>The corrections have been made on lines 124-125 and thank you for detecting that discretion.<br>A paragraph has been added in the discussion regarding manatees rescues in Brazil, which is the next largest program involved with manatees. This discussion does provide a stark contrast to the situation we see in Florida and I do think this inclusion is a good improvement.<br>The last points from Reviewer 3 are exactly the questions we hope this manuscript raises and we are delighted to see this response. We would like to simply pose these questions outright in the manuscript with some thoughts and leave it unanswered as an invitation or encouragement for this work to continue.<br><br>Sincerely<br>Ray L Ball
**Additional Information:**	
**Question**	**Response**
**Financial Disclosure**<br><br>Enter a financial disclosure statement that describes the sources of funding for the work included in this submission. Review the submission guidelines for detailed requirements. View published research articles from *PLOS ONE* for specific examples.<br><br>This statement is required for submission and **will appear in the published article** if the submission is accepted. Please make sure it is accurate.	The author(s) received no specific funding for this work.

**Unfunded studies**<br>Enter: *The author(s) received no specific funding for this work.*<br><br>**Funded studies**<br>Enter a statement with the following details:<br>• Initials of the authors who received each award<br>• Grant numbers awarded to each author<br>• The full name of each funder<br>• URL of each funder website<br>• Did the sponsors or funders play any role in the study design, data collection and analysis, decision to publish, or preparation of the manuscript?<br>• **NO** - Include this sentence at the end of your statement: *The funders had no role in study design, data collection and analysis, decision to publish, or preparation of the manuscript.*<br>• **YES** - Specify the role(s) played.<br><br>* typeset	
**Competing Interests**<br><br>Use the instructions below to enter a competing interest statement for this submission. On behalf of all authors, disclose any competing interests that could be perceived to bias this work—acknowledging all financial support and any other relevant financial or non-financial competing interests.<br><br>This statement **will appear in the published article** if the submission is accepted. Please make sure it is accurate. View published research articles from *PLOS ONE* for specific examples.	The authors have declared that no competing interests exist.

Powered by Editorial Manager® and ProduXion Manager® from Aries Systems Corporation

**NO authors have competing interests**<br><br>Enter: *The authors have declared that no competing interests exist.*<br><br>**Authors with competing interests**<br><br>Enter competing interest details beginning with this statement:<br><br>*I have read the journal's policy and the authors of this manuscript have the following competing interests: [insert competing interests here]*<br><br>* typeset	
**Ethics Statement**<br><br>Enter an ethics statement for this submission. This statement is required if the study involved:<br><br>• Human participants<br>• Human specimens or tissue<br>• Vertebrate animals or cephalopods<br>• Vertebrate embryos or tissues<br>• Field research<br><br>Write "N/A" if the submission does not require an ethics statement.<br><br>General guidance is provided below. Consult the submission guidelines for detailed instructions. **Make sure that all information entered here is included in the Methods section of the manuscript.**	N/A

**Format for specific study types**<br><br>**Human Subject Research (involving human participants and/or tissue)**<br>• Give the name of the institutional review board or ethics committee that approved the study<br>• Include the approval number and/or a statement indicating approval of this research<br>• Indicate the form of consent obtained (written/oral) or the reason that consent was not obtained (e.g. the data were analyzed anonymously)<br><br>**Animal Research (involving vertebrate animals, embryos or tissues)**<br>• Provide the name of the Institutional Animal Care and Use Committee (IACUC) or other relevant ethics board that reviewed the study protocol, and indicate whether they approved this research or granted a formal waiver of ethical approval<br>• Include an approval number if one was obtained<br>• If the study involved *non-human primates*, add *additional details* about animal welfare and steps taken to ameliorate suffering<br>• If anesthesia, euthanasia, or any kind of animal sacrifice is part of the study, include briefly which substances and/or methods were applied<br><br>**Field Research**<br><br>Include the following details if this study involves the collection of plant, animal, or other materials from a natural setting:<br>• Field permit number<br>• Name of the institution or relevant body that granted permission	
**Data Availability**<br><br>Authors are required to make all data underlying the findings described fully available, without restriction, and from the time of publication. PLOS allows rare exceptions to address legal and ethical concerns. See the PLOS Data Policy and FAQ for detailed information.	Yes - all data are fully available without restriction

A Data Availability Statement describing where the data can be found is required at submission. Your answers to this question constitute the Data Availability Statement and **will be published in the article**, if accepted.<br><br>**Important:** Stating 'data available on request from the author' is not sufficient. If your data are only available upon request, select 'No' for the first question and explain your exceptional situation in the text box.<br><br>Do the authors confirm that all data underlying the findings described in their manuscript are fully available without restriction?	
**Describe where the data may be found in full sentences. If you are copying our sample text, replace any instances of XXX with the appropriate details.**<br><br>• If the data are **held or will be held in a public repository**, include URLs, accession numbers or DOIs. If this information will only be available after acceptance, indicate this by ticking the box below. For example: *All XXX files are available from the XXX database (accession number(s) XXX, XXX.*).<br>• If the data are all contained **within the manuscript and/or Supporting Information files**, enter the following: *All relevant data are within the manuscript and its Supporting Information files.*<br>• If neither of these applies but you are able to provide **details of access elsewhere**, with or without limitations, please do so. For example:<br><br>*Data cannot be shared publicly because of [XXX]. Data are available from the XXX Institutional Data Access / Ethics Committee (contact via XXX) for researchers who meet the criteria for access to confidential data.*<br><br>*The data underlying the results presented in the study are available from (include the name of the third party*	All relevant data are within the manuscript and its Supporting Information files.

and contact information or URL).<br>• This text is appropriate if the data are owned by a third party and authors do not have permission to share the data.<br><br>* typeset	
Additional data availability information:	

Powered by Editorial Manager® and ProduXion Manager® from Aries Systems Corporation

Manuscript Click here to access/download;Manuscript;Manuscript.docx

Full title: Trends of the Florida manatee (*Trichechus manatus latirostris*) rehabilitation admissions 1991-2017.

Short title: Florida manatee (*Trichechus manatus latir[illegible]ris*) rehabilitation epidemiology.

Ray L. Ball, [1*#¶], Markuu Malmi, [2¶], Janice Zgibor, [2¶]

[1] ZooTampa at Lowry Park, Department of Medical Sciences, 110 W Sligh Avenue Tampa, FL, 33604, USA

[2] University of South Florida, College of Public Health, Tampa, FL 33620 USA

#Current address: Visiting Assistant Professor, Department of Biology, Eckerd College, St. Petersburg, Florida, 33711, USA

* Corresponding author

E-mail: ballrl@eckerd.edu

¶These authors contributed equally to this work

Abstract

A retrospective study of admission data of 401 West Indian manatees (*Trichechus manatus latirostris)* presented to the David A. Straz Jr. Manatee Critical Care Center at ZooTampa at Lowry Park (ZooTampa) for rehabilitation from August 1991 through October 2017. Causes of admittance, location of rescue, gender, and age class were all recorded for each manatee admitted. Admittance categories as defined by the Florida Fish and Wildlife Conservation Commission (FWC) included watercraft collisions, natural causes, entanglement, entrapment, orphaned calves, captive born, mothers of rescued calves, calves of rescued mothers, human, and other. The admitted population was primarily from the southwest and northwest coasts and related waterways of Florida. The gender difference was relatively equivocal (54% female) while the adults comprised 79% of the admissions. The overall total admissions increased steadily over the study period as did the admissions for each individual categories of admission. Watercraft collisions and natural causes combined were 71% of all admissions for the entire study period and are the dominant causes of admission. Watercraft collisions are more likely to occur during May through October, whereas natural causes of admittance are more likely to occur between December and March. Rehabilitated manatees may reduce overall manatee mortality and can provide insight into population-based health concerns if evaluated appropriately. Future efforts can incorporate physical examination findings, hematology, biochemistry profiles, and ancillary diagnostic testing to continue to improve the individual welfare of this marine mammal in its natural range. Admissions data could also potentially serve the wider conservation and recovery efforts if it is proven that the data obtained is at least as informative as that obtained by the carcass salvage program. Limited conservation resources

could then be re-directed as new challenges arise with the expanding population and potentially expanding range of this species.

Introduction

The West Indian manatee is comprised of two subspecies; the Antillean (*Trichechus manatus manatus)* and the Florida manatee (*Trichechus manatus latirostris).* Watercraft-related deaths and potential loss of warm water refuges are the primary threat to manatee populations [1]. In addition to these threats, drowning due to canal locks and flood gates, entanglement in fishing gear, cold exposure, red tide outbreaks, and habitat loss have all contributed manatee morbidity and mortality and necessitated manatee rescues. The Marine Mammal Protection Act of 1972 [2], Endangered Species Act of 1973 [3], and the Florida Manatee Sanctuary Act of 1978 [4] prohibit any killing, capture, or inhumane harassment of manatees. The West Indian manatee Recovery Plan [5] was implemented in March 1980 and provided a framework to provide protection of this species. As a result of actions such as enforcement of manatee speed zones in waterways, providing manatee sanctuaries, restoration of aquatic vegetation, and education on manatee conservation, the population of the Florida manatee steadily climbed. In 1991, there were an estimated 1,267 Florida manatees, whereas in early 2017 the population was estimated at 6,620 [6]. As of March 30, 2017, the endangered status of the West Indian manatee has been changed to “Threatened” by the United States Fish and Wildlife Service (FWS) under the Endangered Species Act [7]. This change does not affect other federal and state protections afforded manatees.

There are four manatee management units in Florida which include the Upper St. John’s River with an estimated 4% of the population, the Atlantic Coast with 46% of the population, Southwest Florida with 38% of the population, and Northwest Florida with 12% of the population. ZooTampa, Miami Sea Aquarium, Sea World Orlando, and Jacksonville Zoo, are federally permitted critical care facilities for manatee rehabilitation. Identifying the effectiveness of the rehabilitation efforts is essential to all rehabilitation programs and the foundation for improved

release rates, enhanced welfare, and optimal use of resources. This analysis of manatee admittance data at ZooTampa seeks to identify trends of admission rates and locations of rescue stratified by cause of admittance, age, and gender between January 1991 and October 2017. Preliminary analysis of the admittance data may also be useful to predict trends within the population, especially if the rehabilitation data on individual admissions can be summarized and parallels the trends seen in the wild population.

Materials and Methods

ZooTampa, formerly Lowry Park Zoo, has been rehabilitating injured and distressed Florida manatees since August 1991. Manatees that have obvious injuries or are exhibiting abnormal behaviors such as unusual buoyancy and lethargy are typically reported to FWC. Biologists are then dispatched to investigate and determine the need for intervention. Manatees determined to need medical assistance are captured by FWC teams and transported to one of the four federally permitted rehabilitation centers in Florida. Because qualified, practicing veterinarians are not typically on the rescue site nor are they transporting manatees, no medical support is provided until arrival at a critical care facility. On arrival at ZooTampa, manatees are triaged, baseline data is collected including blood sampling, lifesaving procedures are performed if indicated, and the vast majority of the manatees are hospitalized. The ZooTampa manatee medical database from August of 1991 through October 2017 was reviewed and included a total of 429 manatees. Data collection ended in October 2017 when the manatee care center underwent major renovations. Twenty-eight manatees were excluded due to incomplete data. Study variables included gender, age class, cause of admittance, and location of rescue. Mortality, release, and

days in hospital were also collected but are not reported here as they are related to outcomes. Admittance categories as well as causes of death are defined by the Florida Fish and Wildlife Conservation Commission and include watercraft collisions, natural causes (cold stress, brevetoxicosis, anything non-human related), other human causes (entanglement, entrapment, captive born~~,~~ or other causes) and orphaned calves. Mothers of rescued calves and calves of rescued mothers were included in the appropriate category of the manatee requiring rehabilitation. Rehabilitated orphaned manatees must obtain 200cm before being qualified for release. Straight length criteria for manatees is utilized to categorize various life stages ~~of manatees by the biologist~~. Calves are classified as < 235cm, sub-adults from 235 to 265cm~~,~~ and adults > 265cm [8]. A criteria of 200cm was chosen for this study as that straight length is a determinant for both rescue and release criteria. Orphaned calves were defined for this study as calves less than 200cm straight length. Any isolated manatee less than 200cm is considered a dependent calf and will be rescued if possible. Calves undergoing rehabilitation must be 200cm before being considered for release.

FWC manages the manatee rescue and carcass salvage program and divides the Florida into 5 sections (Fig 1); Northeast (NE), East Coast (EC), Southeast (SE), Southwest (SW), and Northwest (NW). The Crystal River (CR) in Citrus County is geographically within the Northwest region but due to the density of manatees in this area and the growing human population, data is recorded for this area separately. Relationships between admittance categories, gender, age class~~,~~ and rescue location were determined using statistical analysis system (SAS).

Figure 1 inserted here.

Results

Table 1 summarizes the gender and age stratification of the manatees admitted during the study period. Adults comprised 79% of the admissions with calves accounting for 21%. The vast majority of these calves were orphans, but injured calves were occasionally admitted with injuries along with their healthy dams (n=3) as well as healthy calves with injured dams (n=13). Females of all ages accounted for 54% of the admissions and males of all ages comprised 46% of admissions.

	Male	Female	Total	% Population of total
Adult	138	177	315	79
Calf	46	40	86	21
Total	184	217	401	
% Population of total	46	54		

Table 1. Manatee admissions data at ZooTampa Augus[illegible]91 to October 2017.

Fig 2 summarizes the admissions data over the course of the study. Admittance was lowest in 1995 having only 1 admission and highest in 2013 with 34 manatees admitted. Fig 3 demonstrates the percentage of admissions by cause. Within the admittance categories for the entire study period, watercraft collisions were the highest with 36.16% of all admissions, followed by natural causes at 34.91%. The third highest category was orphaned calves at 12.7%. No other individual categories had over 5% and they are represented here in one category, other human causes.

Insert Figure 2 here

Insert Figure 3 here

Figure 4 summarizes the trends in cause of admittance in 5-year time blocks over the study period. The last period, starting in 2011, was extended to the end of the study period when the

rehabilitation center was closed. During this period an important change in the cause of admissions was noted with watercraft collisions becoming the dominant cause of admissions. In addition to an overall increase in admissions, each cause of admittance also tended to increase over the study period. Fig 5 demonstrates monthly admissions for the same time periods as in Fig 4. A consistent seasonal variation in total admittance was noted with the highest rates of admittance from January through April. Fig 6 demonstrates that there was also seasonal variation in peaks of admittance due to natural causes in the months of December through April, watercraft collisions May through August, and watercraft again October thru November. Admission of orphaned calves peaks in September and again in December. Orphaned calves comprise 45% of all the admitted calves.

Insert Figure 4 here

Insert figure 5 here

Insert Figure 6 here

Table 2 details the geographical demarcation of the recovery units and the associated percentages of manatee admissions from each region. Rescues and subsequent admissions primarily come from the coast of the Gulf of Mexico and related waterways. More specifically,

the southwest and northwest coasts of Florida, including the Crystal River area, comprise just over 90% of all admissions.

Location of Rescue	No. of manatees	% of total
Northwest	127	31.7
Southwest	215	53.7
East Coast	18	4.4
Northeast	10	2.4
Southeast	6	1.5
Crystal River	21	5.3
Captive Born	4	1.0
Total	401	100

Table 2. Number of manatees admitted to ZooTampa by region over the study period.

The two dominant causes of admission, natural and watercraft were then compared to each other and are summarized in Table 3.

**Variable**	**Odds Ratio**	**95%CI**		**p-value**
Adult vs. Calf	1.26	0.29	5.53	0.76
Female vs. Male	1.78	0.98	3.24	0.06
Location of Rescue				
CR vs. SW	0.67	0.14	3.28	0.63

EC vs. SW	0.20	0.03 1.53	0.13
NE vs. SW	0.28	0.04 2.07	0.22
NW vs. SW	1.20	0.42 3.46	0.73
SE vs. SW	1.29	0.08 21.21	0.86
Years			
1991-1995 vs. 2011-2017	3.12	0.25 38.83	0.38
1996-2000 vs. 2011-2017	0.78	0.26 2.27	0.64
2001-2005 vs. 2011-2017	0.49	0.19 1.24	0.13
2006-2010 vs. 2011-2017	0.90	0.39 2.07	0.80

NW = Northwest: SW = Southwest; EC=East Coast; NE=Northeast; SE=Southeast; CR=Crystal River; CB=Captive Born

Table 3. Comparison between age, gender, location, and 5-year periods between manatees admitted due to natural cause vs watercraft.

Discussion

The Florida manatee population has climbed from 1,267 in 1991 to 6,620 in 2017 and estimates are now as high as 10,280 [9]. This population recovery will inevitably lead to more human-manatee conflicts and it is expected to be in the form of watercraft collisions as noted in the most recent period of this study. Watercraft collisions have played a role in admittance rates in every year of the analysis. Natural causes didn't start having an impact until 1996 when a significant mortality event due to blooms of the dinoflagellate *Karenia brevis*, which resulted in brevetoxicosis [10]. Natural causes from that point forward continued to increase as a cause for admittance and include several significant cold stress related events. The shift in admissions from natural causes to watercraft noted in the recent years may reflect this increased human-manatee interaction. Even with varied admission rates on an annual basis, the overall trend in admissions, both total and cause-specific, has been rising. The sustained trend in rising admissions over this extended period suggests a continued rise in admissions in the future.

The majority of those presented for rehabilitation at ZooTampa come primarily from the west coast, specifically southwest, which isn't surprising given the location of the facility. The northwest rescues did increase around the year 1999. This area includes the Crystal River system with a dense population of manatees. Possible causes could include increased use of an environmental resource, warm water, and/or a larger surveillance of this area. There was no significant difference in the measured parameters when comparing the two most dominant admission categories, natural and watercraft, to each other as seen in Table 3. No attributable risk could be assigned to any measured parameter when comparing the two causes of admissions.

The overall goal of obtaining and analyzing this data was to identify any trends in the admissions to better facilitate management practices to increase rehabilitation recovery rates. Confirming any seasonal tendencies in admission categories has practical applications in the resource management of the rehabilitation hospital and can reinforce public awareness campaigns regarding causes of human-related harm to manatees. Comparisons to all reported manatee rescue events with subsequent admissions for rehabilitation by the various wildlife agencies could prove the admissions data to serve as a useful proxy in the future in the event that manatees continue to be down-listed and rescue recovery efforts are streamlined or if the carcass salvage program is determined to no longer be valuable. The humanitarian effort could continue manatee rescue and rehabilitation, with its associate data collection replacing the data derived from the carcass salvage program. The data integrity could prove to be equivalent. Wildlife rehabilitation can serve as sentinels of wildlife health [11] and perhaps this eventually could be the sentinel method for Florida manatees.

This initial analysis serves as a baseline and template for future analysis. Examining outcomes is the next step in an overall evaluation of rehabilitation efforts in manatees at a single

facility. Mortality data, as well as details of hospitalization time, are also crucial to analyze in any rehabilitation program. Additional recommendations based on medical findings from admissions such as physical examination, hematology and serum biochemistry, and ancillary diagnostics can provide better point of care potential. This could lead to both diagnosis and even therapy on site of rescue, reducing or even eliminating some hospital admissions. Several specific causes of admissions, such as cold stress syndrome [12,13] and brevetoxicosis [14], have some initial data analysis with recommendations being made regarding new potential therapies for both syndromes. As a result of this work, atropine has been recommended for use in brevetoxicosis [14] and anticoagulants for cold stress syndrome [13]. Combining data sets from all the qualified manatee rehabilitation centers could add insight to trends noted here or point out any potential regional differences in terms of admission patterns.

Manatees are also of concern in all parts of their natural range and conservation efforts are in place in various degrees in each location. Brazil has a significant rescue and rehabilitation effort in place and has also reported on the long term efforts. In the Northeastern Aquatic Mammal Stranding Network's territory of Brazils Atlantic coast, an average of three Antillean manatees (*Trichechus manatus manatus*) per year have been rescued from 1987 to 2015 for a total of 77 animals [15]. The mean straight length of these alive recused manatees was 136cm and hence calves. While calves are a significant proportion of manatees rescued in the present study on the Gulf of Mexico coast of Florida, it is vastly different than the scenario described in Brazil. The main threat to this region of Brazil is associated with fisheries and calves becoming stranded [15].

Several intriguing questions are raised that are outside the intended scope of this preliminary work that deserve attention. The rise in watercraft collisions are obvious but is this

the result f the manatee population recovering, a dramatic increase in the number of boats on Florida waterways, or perhaps some combination of both. This situation does have a parallel with the situation involving Florida panthers (*Puma concolor coryi*) and the increase of road killed panthers as the population in total grows. Corroborating the results of the carcass recovery program against this study is a logical step that should be undertaken but again outside the intended scope of this preliminary work. Outcomes, release or death, are the natural progression to this review and their possible impact to the conservation status of manatees should be explored both within this study population and ideally in all manatee populations worldwide.

Acknowledgements

Numerous veterinarians, veterinary technicians, and animal care staff have been involved with the success of the manatee rehabilitation program throughout its history at ZooTampa. We are also grateful for the efforts of the veterinary nursing staff of Heather Henry, Michelle Devlin, and Ryan O'Shea.

References

1. Runge MC, Sanders-Reed CA, Langtimm CA, Fonnesbeck CJ. 2007. A quantitative threats analysis for the Florida manatee (Trichechus manatus latirostris). U.S. Geological Survey. Open-File Report 2007-1086. 34 pp.
2. Marine Mammal Protection Act. 16 USC 1361-1407. https://www.fws.gov/international/pdf/legislation-marine-mammal-protection-act.pdft
3. Endangered Species Act of 1973. https://www.fws.gov/endangered/esa-library/pdf/ESAall.pdf
4. The Florida Manatee Sanctuary Act. Florida Administrative Code & Florida Administrative Register. Rule chapter 68C-22. https://www.flrules.org/gateway/ChapterHome.asp?Chapter=68C-22

5. Brownell, R. West Indian Manatee Recovery Plan. https://www.fws.gov/northflorida/Manatee/Recovery%20Plan/1980_FWS_West_Indian_Manatee_Recovery_Plan.pdf
6. Manatee Synoptic Surveys. https://myfwc.com/research/manatee/research/population-monitoring/synoptic-surveys/
7. Department of the Interior, Fish and Wildlife Service. Endangered and Threatened Wildlife and Plants; Reclassification of the West Indian Manatee From Endangered to Threatened 50 CFR Part 17. RIN 1018-AY84. https://www.fws.gov/southeast/pdf/west-indian-manatee-reclassification-final-rule.pdf
8. Bonde R, Garrett A, Berlanger M, Askin N, Tan L, Wittnich C. Biomedical health assessments of the Florida manatee in Crystal River: Providing opportunities for training during the capture, handling, and processing of this unique aquatic mammal. *Journal of Marine Animals and Their Ecology*, *5*(2), 17-28. (2012b).
9. Hostettler J. Updated statewide abundance estimates for the Florida manatee 2018 https://f50006a.eos-intl.net/F50006A/OPAC/Details/Record.aspx?BibCode=1864664
10. Bossart G, Baden D, Ewing R, Roberts B, Wright D. Brevetoxicosis in manatees (*Trichechus manatus latirostris*) from the 1996 epizootic: gross, histologic, and immunohistochemical features. Toxicologic Pathology 1998 Mar-Apr; Vol. 26 (2), pp. 276-82.
11. Randall N, BlitvichB, BlanchongJ. Efficacy of wildlife rehabilitation centers in surveillance and monitoring of pathogen activity: a case study with West Nile virus. J Wildl Dis. 2012 Jul;48(3):646-53.
12. Ball R. Thromboembolic Disease as a Component of Health Issues in the Florida Manatee (*Trichechus manatus latirostris*). Proceeding of IAAAM 2013. https://www.vin.com/apputil/content/defaultadv1.aspx?pId=11375&meta=Generic&id=5768576
13. Gerlach T, Bandt C, Conner B, Ball R. Journal of the American Veterinary Medical Association. 11/1/2015, Vol. 247 Issue 9, p1048-1065. 8p. DOI: 10.2460/javma.247.9.1048.
14. Ball R, Walsh C, Flewelling L, Arnett L, Smith L et al. Clinical Pathology, Serum Brevetoxin, and Clinical Signs of Florida Manatees (*Trichechus manatus latirostris*) During the Brevetoxin-Related Mortality Event in Southwest Florida 2013. Proceeding IAAAM 2014. https://www.vin.com/apputil/content/defaultadv1.aspx?pId=11397&meta=Generic&id=6251877
15. Balensiefer CD, Attademo F, Sousa G, Freire A, Cunha F, Barboza A, Silva F, and Luna F. Three Decades of Antillean Manatee (Trichechus manatus manatus) Stranding Along the Brazilian Coast. Tropical Conservation Science Volume 10: 1–9. DOI: 10.1177/1940082917728375

Supporting Information

S1. FWC Manatee Rescue and Salvage Program Map.

S2. Manatee admissions over the study period.

S3. Percent of manatees admitted by cause of admittance.

S4. Number of manatees admitted over study period by cause of admission.

S5. Number of manatees admitted each month by year.

S6. Manatees admitted by cause of admittance by month.

 Click here to access/download;Figure;S2. Fig 2 .docx

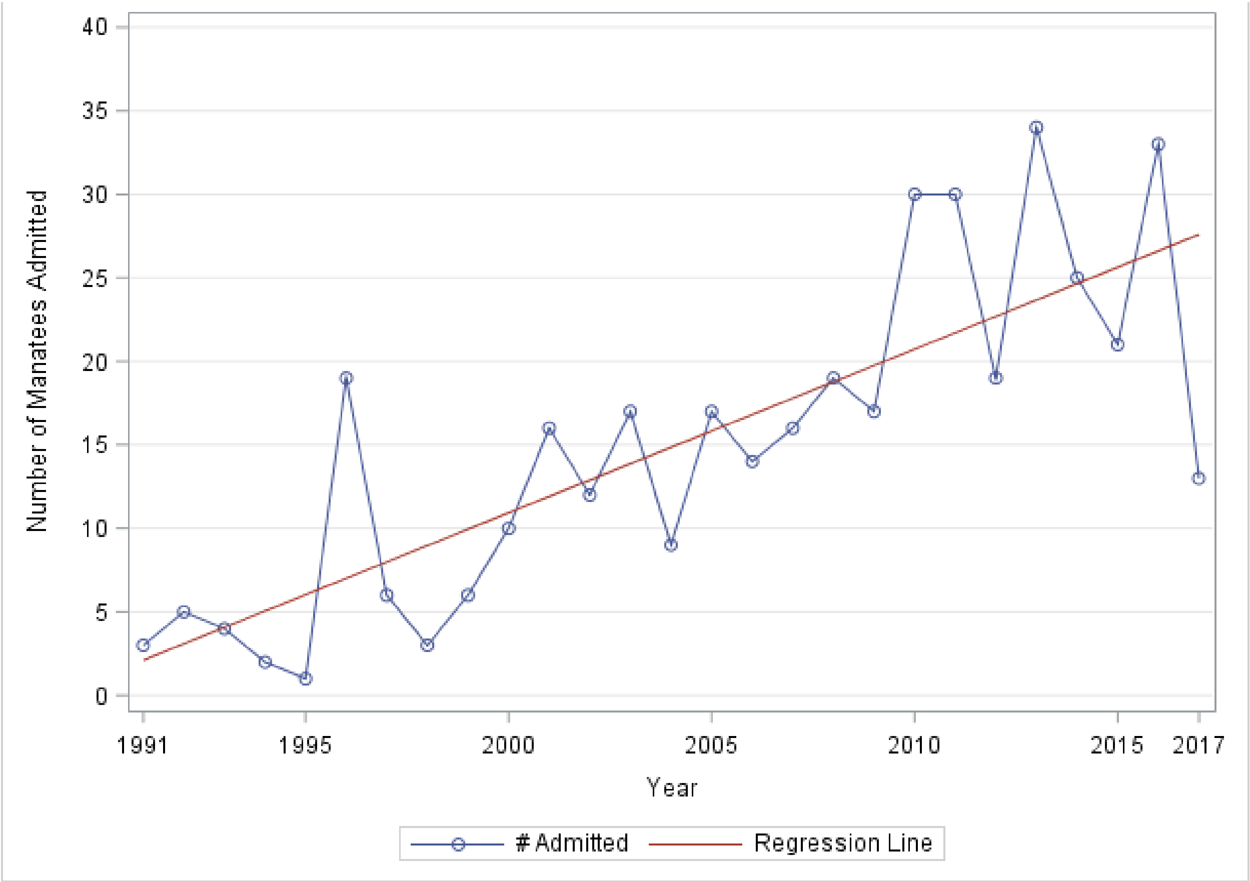

Fig 2. Admittance of manatees at ZooTampa from August 1991 through October 2017.

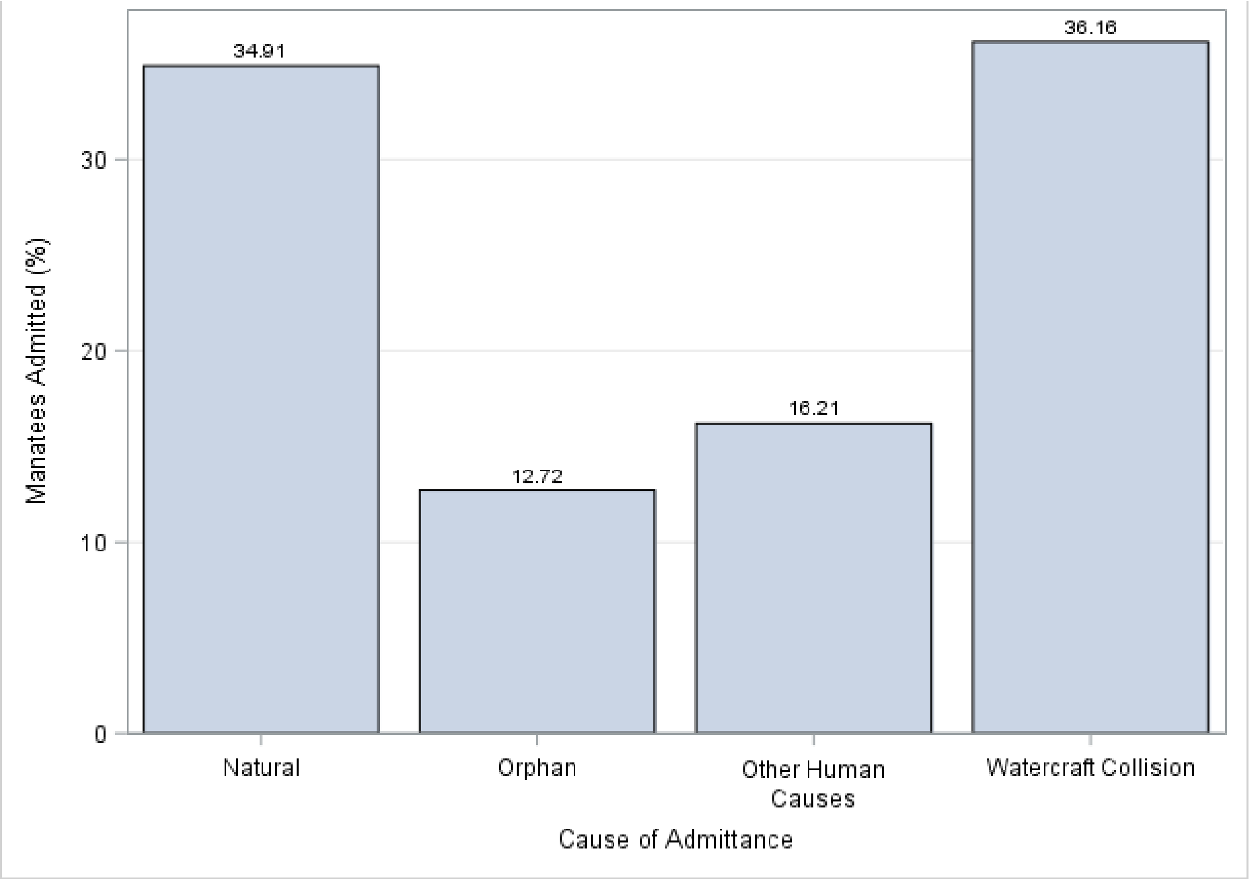

Fig. 3. Percent of manatees admitted at ZooTampa for rehabilitation by cause of admittance

 Click here to access/download;Figure;S4. Fig 4. .docx

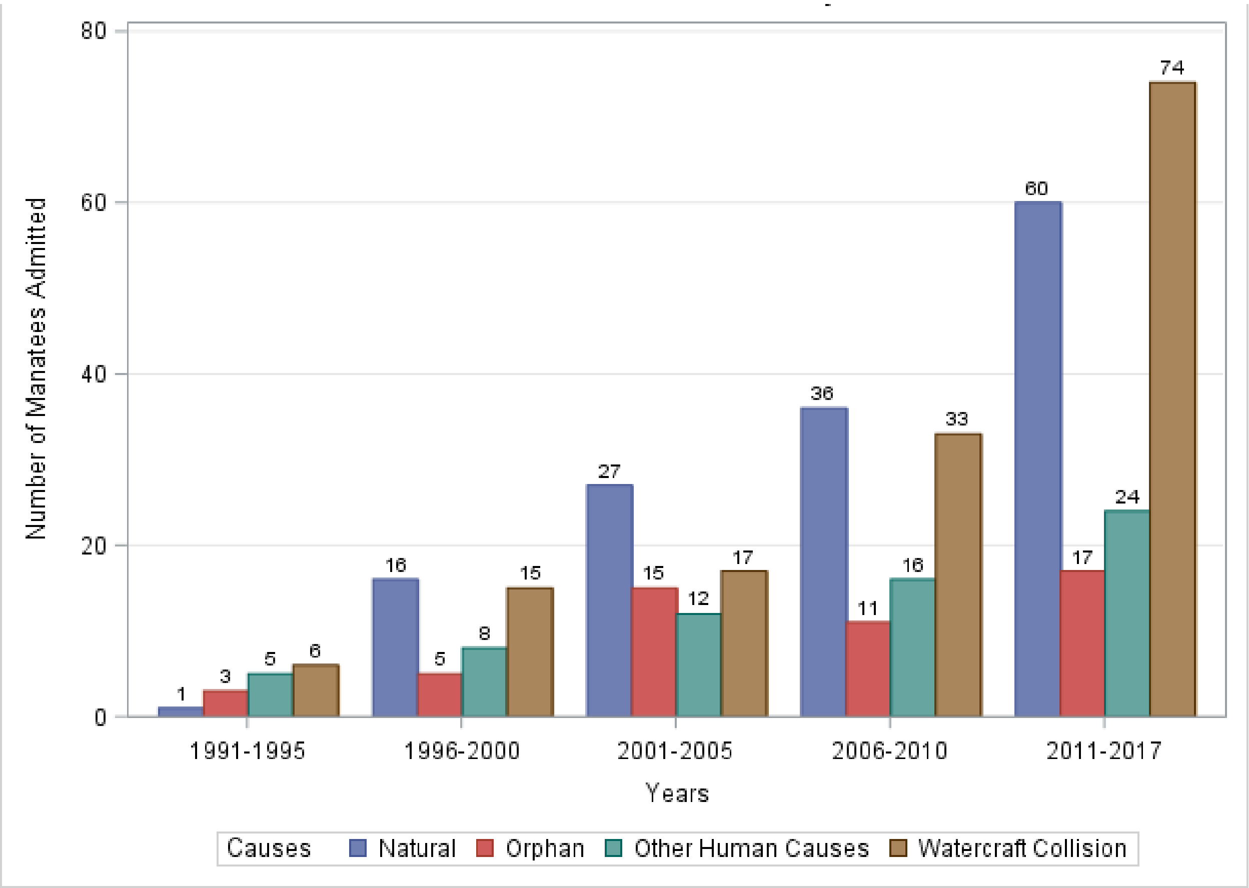

Fig 4. Manatees admitted to ZooTampa over time by cause of admittance

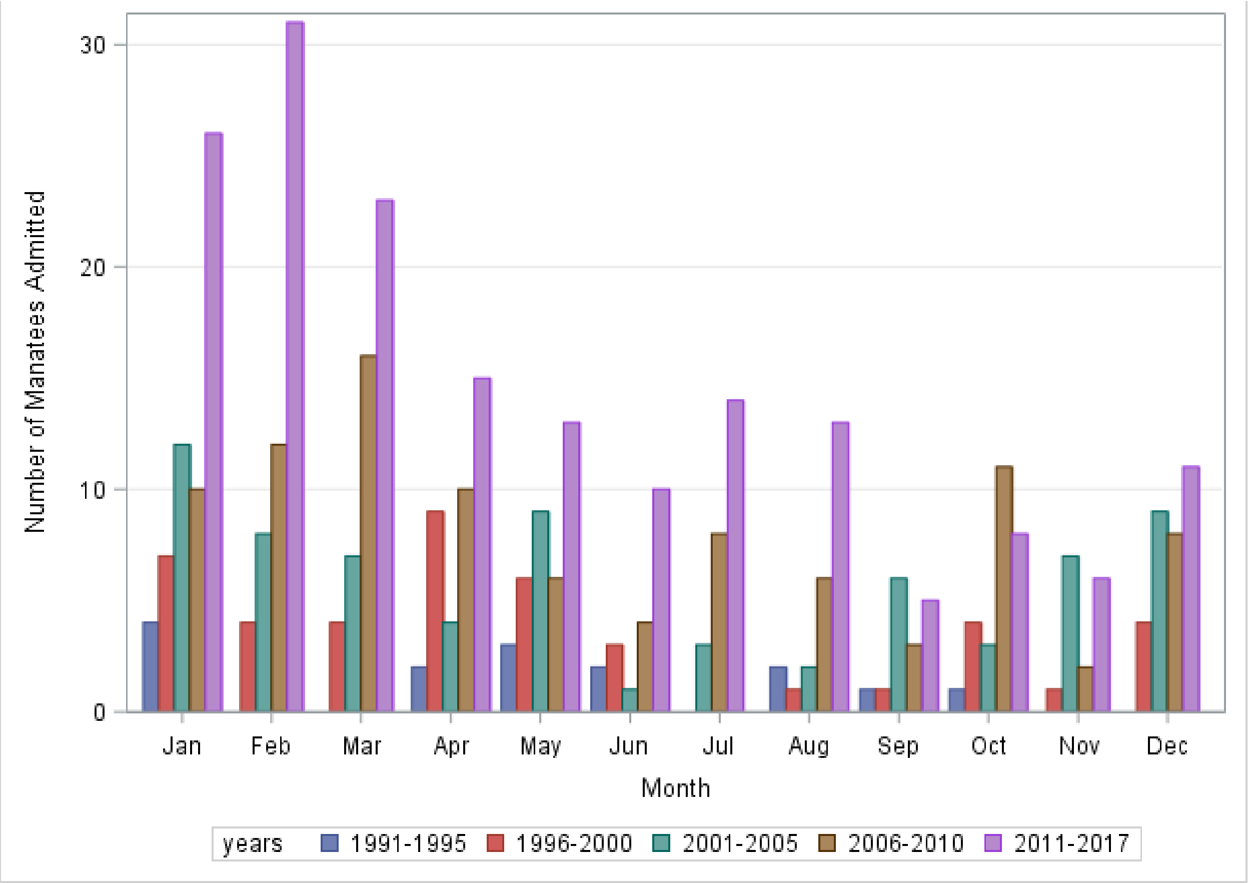

Fig 5. Seasonal variation in admittance of manatees to ZooTampa by month over the study period. January through April tend to be peak period for admissions.

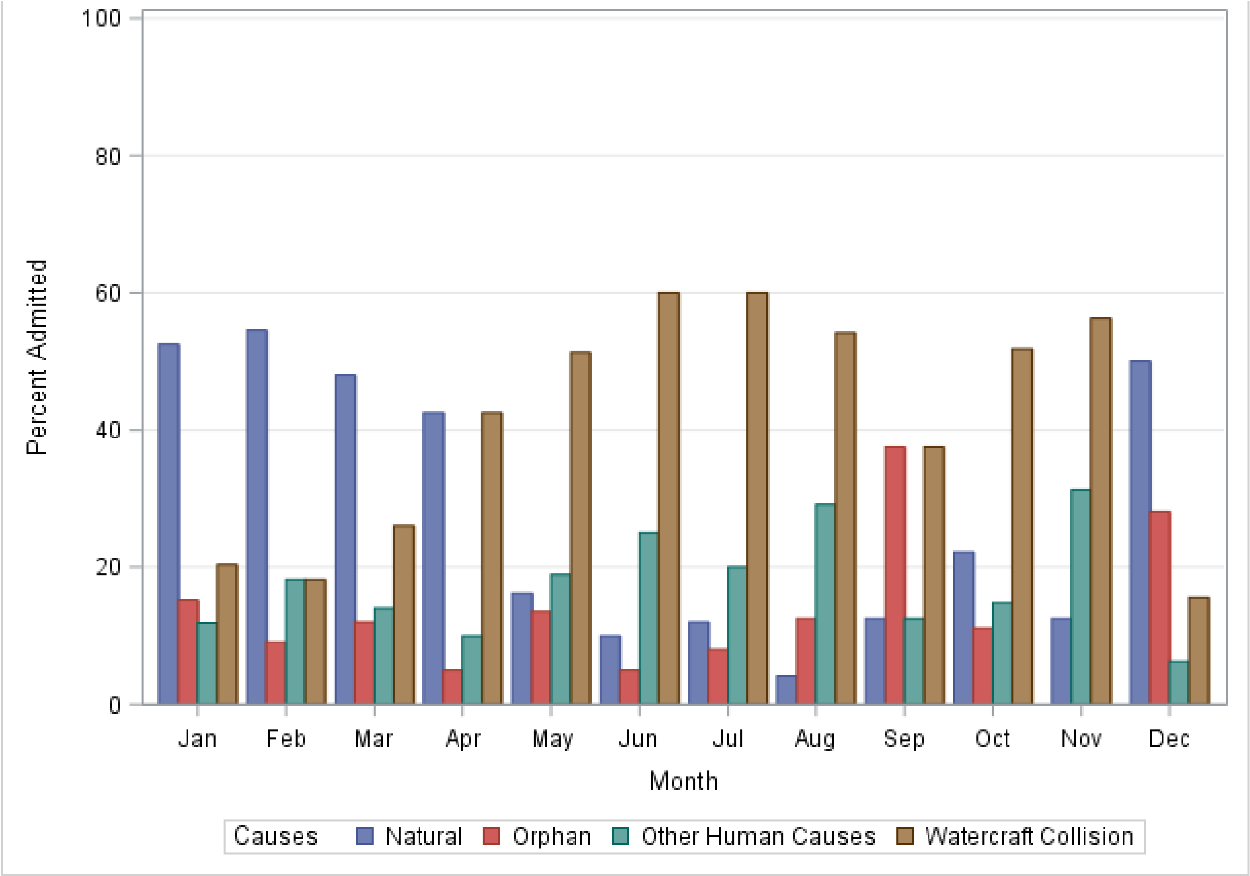

Fig 6. Total manatees admitted by cause summated by month for entire study period.

 Click here to access/download;Figure;S1. Fig 1.docx

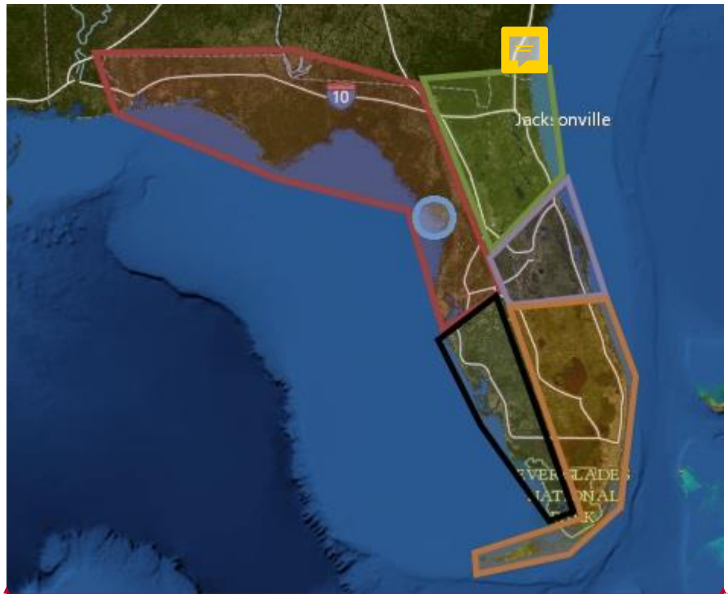

Fig 1. Florida Fish and Wildlife Commission Rescue and Carcass Salvage Program map of Florida. Red outline – Northwest (NW), Green outline – Northeast (NE), Purple outline – East Coast (EC), Orange outline – Southeast (SE), Black outline – Southwest (SW). Blue Circle – Crystal River (CR). Map provided by http://viewer.nationalmap.gov/viewer/

Formatted: Font: (Default) Times New Roman, 12 pt

Formatted: Font: (Default) Times New Roman, 12 pt

Formatted: Font: Times New Roman, 12 pt

Formatted: Font: (Default) Times New Roman, 12 pt

Data

Click here to access/download
Supporting Information
m1.sas7bdat

Full title: Trends of the Florida manatee (*Trichechus manatus latirostris*) rehabilitation admissions 1991-2017.

Short title: Florida manatee (*Trichechus manatus latirostris*) rehabilitation epidemiology.

Ray L. Ball, [1*#¶], Markuu Malmi, [2¶], Janice Zgibor, [2¶]

[1] ZooTampa at Lowry Park, Department of Medical Sciences, 110 W Sligh Avenue Tampa, FL, 33604, USA

[2] University of South Florida, College of Public Health, Tampa, FL 33620 USA

#Current address: Visiting Assistant Professor, Department of Biology, Eckerd College, St. Petersburg, Florida, 33711, USA

* Corresponding author

E-mail: ballrl@eckerd.edu

¶These authors contributed equally to this work

Abstract

A retrospective study of admission data of 401 West Indian manatees (*Trichechus manatus latirostris)* presented to the David A. Straz Jr. Manatee Critical Care Center at ZooTampa at Lowry Park (ZooTampa) for rehabilitation from August 1991 through October 2017. Causes of admittance, location of rescue, gender, and age class were all recorded for each manatee admitted. Admittance categories as defined by the Florida Fish and Wildlife Conservation Commission (FWC) included watercraft collisions, natural causes, entanglement, entrapment, orphaned calves, captive born, mothers of rescued calves, calves of rescued mothers, human, and other. The admitted population was primarily from the southwest and northwest coasts and related waterways of Florida. The gender difference was relatively equivocal (54% female) while the adults comprised 79% of the admissions. The overall total admissions increased steadily over the study period as did the admissions for each individual categories of admission. Watercraft collisions and natural causes combined were 71% of all admissions for the entire study period and are the dominant causes of admission. Watercraft collisions are more likely to occur during May through October, whereas natural causes of admittance are more likely to occur between December and March. Rehabilitated manatees may reduce overall manatee mortality and can provide insight into population-based health concerns if evaluated appropriately. Future efforts can incorporate physical examination findings, hematology, biochemistry profiles, and ancillary diagnostic testing to continue to improve the individual welfare of this marine mammal in its natural range. Admissions data could also potentially serve the wider conservation and recovery efforts if it is proven that the data obtained is at least as informative as that obtained by the carcass salvage program. Limited conservation resources

could then be re-directed as new challenges arise with the expanding population and potentially expanding range of this species.

Introduction

The West Indian manatee is comprised of two subspecies; the Antillean (*Trichechus manatus manatus)* and the Florida manatee (*Trichechus manatus latirostris).* Watercraft-related deaths and potential loss of warm water refuges are the primary threat to manatee populations [1]. In addition to these threats, drowning due to canal locks and flood gates, entanglement in fishing gear, cold exposure, red tide outbreaks, and habitat loss have all contributed manatee morbidity and mortality and necessitated manatee rescues. The Marine Mammal Protection Act of 1972 [2], Endangered Species Act of 1973 [3], and the Florida Manatee Sanctuary Act of 1978 [4] prohibit any killing, capture, or inhumane harassment of manatees. The West Indian manatee Recovery Plan [5] was implemented in March 1980 and provided a framework to provide protection of this species. As a result of actions such as enforcement of manatee speed zones in waterways, providing manatee sanctuaries, restoration of aquatic vegetation, and education on manatee conservation, the population of the Florida manatee steadily climbed. In 1991, there were an estimated 1,267 Florida manatees, whereas in early 2017 the population was estimated at 6,620 [6]. As of March 30, 2017, the endangered status of the West Indian manatee has been changed to “Threatened” by the United States Fish and Wildlife Service (FWS) under the Endangered Species Act [7]. This change does not affect other federal and state protections afforded manatees.

There are four manatee management units in Florida which include the Upper St. John’s River with an estimated 4% of the population, the Atlantic Coast with 46% of the population, Southwest Florida with 38% of the population, and Northwest Florida with 12% of the population. ZooTampa, Miami Sea Aquarium, Sea World Orlando, and Jacksonville Zoo, are federally permitted critical care facilities for manatee rehabilitation. Identifying the effectiveness of the rehabilitation efforts is essential to all rehabilitation programs and the foundation for improved

release rates, enhanced welfare, and optimal use of resources. This analysis of manatee admittance data at ZooTampa seeks to identify trends of admission rates and locations of rescue stratified by cause of admittance, age, and gender between January 1991 and October 2017. Preliminary analysis of the admittance data may also be useful to predict trends within the population, especially if the rehabilitation data on individual admissions can be summarized and parallels the trends seen in the wild population.

Materials and Methods

ZooTampa, formerly Lowry Park Zoo, has been rehabilitating injured and distressed Florida manatees since August 1991. Manatees that have obvious injuries or are exhibiting abnormal behaviors such as unusual buoyancy and lethargy are typically reported to FWC. Biologists are then dispatched to investigate and determine the need for intervention. Manatees determined to need medical assistance are captured by FWC teams and transported to one of the four federally permitted rehabilitation centers in Florida. Because qualified, practicing veterinarians are not typically on the rescue site nor are they transporting manatees, no medical support is provided until arrival at a critical care facility. On arrival at ZooTampa, manatees are triaged, baseline data is collected including blood sampling, lifesaving procedures are performed if indicated, and the vast majority of the manatees are hospitalized. The ZooTampa manatee medical database from August of 1991 through October 2017 was reviewed and included a total of 429 manatees. Data collection ended in October 2017 when the manatee care center underwent major renovations. Twenty-eight manatees were excluded due to incomplete data. Study variables included gender, age class, cause of admittance, and location of rescue. Mortality, release, and

days in hospital were also collected but are not reported here as they are related to outcomes. Admittance categories as well as causes of death are defined by the Florida Fish and Wildlife Conservation Commission and include~~d~~ watercraft collisions, natural causes (cold stress, brevetoxicosis, anything non-human related), other human causes (entanglement, entrapment, captive born, or other causes) and orphaned calves. Mothers of rescued calves and calves of rescued mothers were included in the appropriate category of the manatee requiring rehabilitation. Rehabilitated orphaned manatees must obtain 200cm before being qualified for release. Straight length criteria for manatees is utilized to categorize various life stages of manatees by the biologist. Calves are classified as < 235cm, sub-adults from 235 to 265cm, and adults > 265cm [8]. A criteria of 200cm was chosen for this study as that straight length is a determinant for both rescue and release criteria. Orphaned calves were defined for this study as calves less than 200cm straight length. Any isolated manatee less than 200cm is considered a dependent calf and will be rescued if possible. Calves undergoing rehabilitation must be 200cm before being considered for release.

FWC manages the manatee rescue and carcass salvage program and divides the Florida into 5 sections (Fig 1); Northeast (NE), East Coast (EC), Southeast (SE), Southwest (SW), and Northwest (NW). The Crystal River (CR) in Citrus County is geographically within the Northwest region but due to the density of manatees in this area and the growing human population, data is recorded for this area separately. Relationships between admittance categories, gender, age class, and rescue location were determined using statistical analysis system (SAS).

Figure 1 inserted here.

Results

Table 1 summarizes the gender and age stratification of the manatees admitted during the study period. Adults comprised 79% of the admissions with calves accounting for 21%. The vast majority of these calves were orphans, but injured calves were occasionally admitted with injuries along with their healthy dams (n=3) as well as healthy calves with injured dams (n=13). Females of all ages accounted for 54% of the admissions and males of all ages comprised 46% of admissions.

	Male	Female	Total	% Population of total
Adult	138	177	315	79
Calf	46	40	86	21
Total	184	217	401	
% Population of total	46	54		

Table 1. Manatee admissions data at ZooTampa August 1991 to October 2017.

Fig 2 summarizes the admissions data over the course of the study. Admittance was lowest in 1995 having only 1 admission and highest in 2013 with 34 manatees admitted. Fig 3 demonstrates the percentage of admissions by cause. Within the admittance categories for the entire study period, ~~natural causes~~watercraft collisions ~~-~~were the highest with 36.~~2~~16% of all admissions, followed by ~~watercraft collisions~~natural causes at 34.91%. The third highest category was orphaned calves at 12.7%. No other individual categories had over 5% and they are represented here in one category, other human causes.

Insert Figure 2 here

Insert Figure 3 here

Figure 4 summarizes the trends in cause of admittance in 5-year time blocks over the study period. The last period, starting in 2011, was extended to the end of the study period when the

rehabilitation center was closed. During this period an important change in the cause of admissions was noted with watercraft collisions becoming the dominant cause of admissions. In addition to an overall increase in admissions, each cause of admittance also tended to increase over the study period. Fig 5 demonstrates monthly admissions for the same time periods as in Fig 4. A consistent seasonal variation in total admittance was noted with the highest rates of admittance from January through April. Fig 6 demonstrates that there was also seasonal variation in peaks of admittance due to natural causes in the months of December through April, watercraft collisions May through August, and watercraft again October thru November. Admission of orphaned calves peaks in September and again in December. Orphaned calves comprise 45% of all the admitted calves.

Insert Figure 4 here

Insert figure 5 here

Insert Figure 6 here

Table 2 details the geographical demarcation of the recovery units and the associated percentages of manatee admissions from each region. Rescues and subsequent admissions primarily come from the coast of the Gulf of Mexico and related waterways. More specifically,

the southwest and northwest coasts of Florida, including the Crystal River area, comprise just over 90% of all admissions.

Location of Rescue	No. of manatees	% of total
Northwest	127	31.7
Southwest	215	53.7
East Coast	18	4.4
Northeast	10	2.4
Southeast	6	1.5
Crystal River	21	5.3
Captive Born	4	1.0
Total	401	100

Table 2. Number of manatees admitted to ZooTampa by region over the study period.

The two dominant causes of admission, natural and watercraft were then compared to each other and are summarized in Table 3.

**Variable**	**Odds Ratio**	**95%CI**	**p-value**
Adult vs. Calf	1.26	0.29 5.53	0.76
Female vs. Male	1.78	0.98 3.24	0.06
Location of Rescue			
CR vs. SW	0.67	0.14 3.28	0.63

EC vs. SW	0.20	0.03 1.53	0.13
NE vs. SW	0.28	0.04 2.07	0.22
NW vs. SW	1.20	0.42 3.46	0.73
SE vs. SW	1.29	0.08 21.21	0.86
Years			
1991-1995 vs. 2011-2017	3.12	0.25 38.83	0.38
1996-2000 vs. 2011-2017	0.78	0.26 2.27	0.64
2001-2005 vs. 2011-2017	0.49	0.19 1.24	0.13
2006-2010 vs. 2011-2017	0.90	0.39 2.07	0.80

NW = Northwest: SW = Southwest; EC=East Coast; NE=Northeast; SE=Southeast; CR=Crystal River; CB=Captive Born

Table 3. Comparison between age, gender, location, and 5-year periods between manatees admitted due to natural cause vs watercraft.

Discussion

The Florida manatee population has climbed from 1,267 in 1991 to 6,620 in 2017 and estimates are now as high as 10,280 [9]. This population recovery will inevitably lead to more human-manatee conflicts and it is expected to be in the form of watercraft collisions as noted in the most recent period of this study. Watercraft collisions have played a role in admittance rates in every year of the analysis. Natural causes didn't start having an impact until 1996 when a significant mortality event due to blooms of the dinoflagellate *Karenia brevis*, which resulted in brevetoxicosis [10]. Natural causes from that point forward continued to increase as a cause for admittance and include several significant cold stress related events. The shift in admissions from natural causes to watercraft noted in the recent years may reflect this increased human-manatee interaction. Even with varied admission rates on an annual basis, the overall trend in admissions, both total and cause-specific, has been rising. The sustained trend in rising admissions over this extended period suggests a continued rise in admissions in the future.

The majority of those presented for rehabilitation at ZooTampa come primarily from the west coast, specifically southwest, which isn't surprising given the location of the facility. The northwest rescues did increase around the year 1999. This area includes the Crystal River system with a dense population of manatees. Possible causes could include increased use of an environmental resource, warm water, and/or a larger surveillance of this area. There was no significant difference in the measured parameters when comparing the two most dominant admission categories, natural and watercraft, to each other as seen in Table 3. No attributable risk could be assigned to any measured parameter when comparing the two causes of admissions.

The overall goal of obtaining and analyzing this data was to identify any trends in the admissions to better facilitate management practices to increase rehabilitation recovery rates. Confirming any seasonal tendencies in admission categories has practical applications in the resource management of the rehabilitation hospital and can reinforce public awareness campaigns regarding causes of human-related harm to manatees. Comparisons to all reported manatee rescue events with subsequent admissions for rehabilitation by the various wildlife agencies could prove the admissions data to serve as a useful proxy in the future in the event that manatees continue to be down-listed and rescue recovery efforts are streamlined or if the carcass salvage program is determined to no longer be valuable. The humanitarian effort could continue manatee rescue and rehabilitation, with its associate data collection replacing the data derived from the carcass salvage program. The data integrity could prove to be equivalent. Wildlife rehabilitation can serve as sentinels of wildlife health [11] and perhaps this eventually could be the sentinel method for Florida manatees.

This initial analysis serves as a baseline and template for future analysis. Examining outcomes is the next step in an overall evaluation of rehabilitation efforts in manatees at a single

facility. Mortality data, as well as details of hospitalization time, are also crucial to analyze in any rehabilitation program. Additional recommendations based on medical findings from admissions such as physical examination, hematology and serum biochemistry, and ancillary diagnostics can provide better point of care potential. This could lead to both diagnosis and even therapy on site of rescue, reducing or even eliminating some hospital admissions. Several specific causes of admissions, such as cold stress syndrome [12,13] and brevetoxicosis [14], have some initial data analysis with recommendations being made regarding new potential therapies for both syndromes. As a result of this work, atropine has been recommended for use in brevetoxicosis [14] and anticoagulants for cold stress syndrome [13]. Combining data sets from all the qualified manatee rehabilitation centers could add insight to trends noted here or point out any potential regional differences in terms of admission patterns.

Manatees are also of concern in all parts of their natural range and conservation efforts are in place in various degrees in each location. Brazil has a significant rescue and rehabilitation effort in place and has also reported on the long term efforts. In the Northeastern Aquatic Mammal Stranding Network's territory of Brazils Atlantic coast, an average of three Antillean manatees (*Trichechus manatus manatus*) per year have been rescued from 1987 to 2015 for a total of 77 animals [15]. The mean straight length of these alive recused manatees was 136cm and hence calves. While calves are a significant proportion of manatees rescued in the present study on the Gulf of Mexico coast of Florida, it is vastly different than the scenario described in Brazil. The main threat to this region of Brazil is associated with fisheries and calves becoming stranded [15].

Several intriguing questions are raised that are outside the intended scope of this preliminary work that deserve attention. The rise in watercraft collisions are obvious but is this

Formatted: Left, Space After: 0 pt, Don't adjust space between Latin and Asian text, Don't adjust space between Asian text and numbers, Pattern: Clear

Formatted: Font: (Default) Times New Roman, 12 pt

Formatted: Font: (Default) Times New Roman, 12 pt, Italic

Formatted: Font: (Default) Times New Roman, 12 pt

the result f the manatee population recovering, a dramatic increase in the number of boats on Florida waterways, or perhaps some combination of both. This situation does have a parallel with the situation involving Florida panthers (*Puma concolor coryi*) and the increase of road killed panthers as the population in total grows. Corroborating the results of the carcass recovery program against this study is a logical step that should be undertaken but again outside the intended scope of this preliminary work. Outcomes, release or death, are the natural progression to this review and their possible impact to the conservation status of manatees should be explored both within this study population and ideally in all manatee populations worldwide.

Acknowledgements

Numerous veterinarians, veterinary technicians, and animal care staff have been involved with the success of the manatee rehabilitation program throughout its history at ZooTampa. We are also grateful for the efforts of the veterinary nursing staff of Heather Henry, Michelle Devlin, and Ryan O'Shea. ~~The McCune and McCann Foundations were instrumental in providing funding for both clinical fellowships and resources to facilitate diagnostic investigations.~~

References

1. Runge MC, Sanders-Reed CA, Langtimm CA, Fonnesbeck CJ. 2007. A quantitative threats analysis for the Florida manatee (Trichechus manatus latirostris). U.S. Geological Survey. Open-File Report 2007-1086. 34 pp.
2. Marine Mammal Protection Act. 16 USC 1361-1407. https://www.fws.gov/international/pdf/legislation-marine-mammal-protection-act.pdft
3. Endangered Species Act of 1973. https://www.fws.gov/endangered/esa-library/pdf/ESAall.pdf
4. The Florida Manatee Sanctuary Act. Florida Administrative Code & Florida Administrative Register. Rule chapter 68C-22. https://www.flrules.org/gateway/ChapterHome.asp?Chapter=68C-22

Formatted: Font: Italic

Formatted: Font: Times New Roman, 12 pt

Formatted: Font: Times New Roman, 12 pt

5. Brownell, R. West Indian Manatee Recovery Plan. https://www.fws.gov/northflorida/Manatee/Recovery%20Plan/1980_FWS_West_Indian_Manatee_Recovery_Plan.pdf
6. Manatee Synoptic Surveys. https://myfwc.com/research/manatee/research/population-monitoring/synoptic-surveys/
7. Department of the Interior, Fish and Wildlife Service. Endangered and Threatened Wildlife and Plants; Reclassification of the West Indian Manatee From Endangered to Threatened 50 CFR Part 17. RIN 1018-AY84. https://www.fws.gov/southeast/pdf/west-indian-manatee-reclassification-final-rule.pdf
8. Bonde R, Garrett A, Berlanger M, Askin N, Tan L, Wittnich C. Biomedical health assess-ments of the Florida manatee in Crystal River: Providing opportunities for training during the capture, handling, and processing of this unique aquatic mammal. *Journal of Marine Animals and Their Ecology*, *5*(2), 17-28. (2012b).
9. Hostettler J. Updated statewide abundance estimates for the Florida manatee 2018 https://f50006a.eos-intl.net/F50006A/OPAC/Details/Record.aspx?BibCode=1864664
10. Bossart G, Baden D, Ewing R, Roberts B, Wright D. Brevetoxicosis in manatees (*Trichechus manatus latirostris*) from the 1996 epizootic: gross, histologic, and immunohistochemical features. Toxicologic Pathology 1998 Mar-Apr; Vol. 26 (2), pp. 276-82.
11. Randall N, BlitvichB, BlanchongJ. Efficacy of wildlife rehabilitation centers in surveillance and monitoring of pathogen activity: a case study with West Nile virus. J Wildl Dis. 2012 Jul;48(3):646-53.
12. Ball R. Thromboembolic Disease as a Component of Health Issues in the Florida Manatee (*Trichechus manatus latirostris*). Proceeding of IAAAM 2013. https://www.vin.com/apputil/content/defaultadv1.aspx?pId=11375&meta=Generic&id=5768576
13. Gerlach T, Bandt C, Conner B, Ball R. Journal of the American Veterinary Medical Association. 11/1/2015, Vol. 247 Issue 9, p1048-1065. 8p. DOI: 10.2460/javma.247.9.1048.
14. Ball R, Walsh C, Flewelling L, Arnett L, Smith L et al. Clinical Pathology, Serum Brevetoxin, and Clinical Signs of Florida Manatees (*Trichechus manatus latirostris*) During the Brevetoxin-Related Mortality Event in Southwest Florida 2013. Proceeding IAAAM 2014. https://www.vin.com/apputil/content/defaultadv1.aspx?pId=11397&meta=Generic&id=6251877
15. Balensiefer CD, Attademo F, Sousa G, Freire A, Cunha F, Barboza A, Silva F, — and Luna F. Three Decades of Antillean Manatee (Trichechus manatus manatus) Stranding Along the Brazilian Coast. Tropical Conservation Science Volume 10: 1–9. DOI: 10.1177/1940082917728375

Supporting Information

S1. FWC Manatee Rescue and Salvage Program Map.

Formatted: Font: Font color: Auto, Small caps

Formatted: Font: (Default) Times New Roman, 12 pt, Font color: Red

Formatted: List Paragraph, Numbered + Level: 1 + Numbering Style: 1, 2, 3, … + Start at: 1 + Alignment: Left + Aligned at: 0.25" + Indent at: 0.5"

S2. Manatee admissions over the study period.

S3. Percent of manatees admitted by cause of admittance.

S4. Number of manatees admitted over study period by cause of admission.

S5. Number of manatees admitted each month by year.

S6. Manatees admitted by cause of admittance by month.

Responses to reviewers for Trends of the Florida manatee (*Trichechus manatus latirostris*) rehabilitation admissions 1991-2017 by Ball, Malmi, and Zgibor.

We would like to thank all the reviewers for their efforts in improving our contribution. We have attempted to address all the issues but do have some differences in opinion in what message we are looking to share at this point. These will be detailed below. The first point is that the statistical analysis has been conducted from an epidemiological perspective by epidemiologist in human public health and we believe they are rigorous and yet descriptive enough to be read and understood by readers of various academic backgrounds. With all due respect, we have elected to not alter these methods for this publication.

As for the funding statement, we were simply trying to acknowledge supporters of the entire rehabilitation program but no specific funding was obtained in the review and preparation of this manuscript. "The funders had no role in study design, data collection and analysis, decision to publish, or preparation of the manuscript."

The causes of admission and death of manatees are defined, as now noted in the manuscript, by the Florida Fish and Wildlife Conservation Commission. In our opinion this reads well and is the vernacular that is used both within our

community and with the public at large when communicating about manatees. We are not sure a table would be useful.

A detailed map showing all the rescue points would indeed be interesting but we believe too detailed for the purposes intended here. Work of this nature would be most appropriate as part of a thesis. The current manuscript is not delving into outcomes of the manatee rehabilitation; that is a follow-up manuscript so we think the descriptors are adequate. It is indeed an important aspect of the entire endeavor but again we are looking to focus our current efforts to the admission and see if there are lessons we can share.

Figures 5 and 6 do look similar but the point of each is distinctly different. Figure 5, with a revised caption, highlights the overall increase in manatee admissions over time in 5 year blocks. Figure 6 highlights the seasonality of admissions based on the admission categories.

We agree that some comparison to manatee rescues outside the USA would be appropriate. Brazil is really the only other country that does this with any frequency and that is the basis of the comparison I have added. It becomes problematic to compare facilities with the USA and within Florida as each facility is a private business, both non-profit and profit making operations. Sharing of medical data can be a challenge on an open basis that would include publication.

One objective of this manuscript is to start to break down that barrier. With the completion of this manuscript, perhaps other facilities will then be open to compile their own information and data can be compared then. The fact that such a review as we are undertaking has *not* taken place yet really speaks volumes. This point is actually mentioned in the manuscript in lines 212 - 214.

The corrections have been made on lines 124-125 and thank you for detecting that discretion.

A paragraph has been added in the discussion regarding manatees rescues in Brazil, which is the next largest program involved with manatees. This discussion does provide a stark contrast to the situation we see in Florida and I do think this inclusion is a good improvement.

The last points from Reviewer 3 are exactly the questions we hope this manuscript raises and we are delighted to see this response. We would like to simply pose these questions outright in the manuscript with some thoughts and leave it unanswered as an invitation or encouragement for this work to continue.
