# Supplementary material for: Trends of the Florida manatee (Trichechus manatus latirostris) rehabilitation admissions 1991-2017
Source: PLoS One. 2020 Jul 9;15(7):e0223207. doi: 10.1371/journal.pone.0223207 (PMC7347118; doi:10.1371/journal.pone.0223207)
Supplement: Attachment — Submitted filename: Response to Reviewers.docx [file pone.0223207.s002.docx]

Responses to reviewers for Trends of the Florida manatee (*Trichechus manatus latirostris*) rehabilitation admissions 1991-2017 by Ball, Malmi, and Zgibor.

We would like to thank all the reviewers for their efforts in improving our contribution. We have attempted to address all the issues but do have some differences in opinion in what message we are looking to share at this point. These will be detailed below. The first point is that the statistical analysis has been conducted from an epidemiological perspective by epidemiologist in human public health and we believe they are rigorous and yet descriptive enough to be read and understood by readers of various academic backgrounds. With all due respect, we have elected to not alter these methods for this publication.

As for the funding statement, we were simply trying to acknowledge supporters of the entire rehabilitation program but no specific funding was obtained in the review and preparation of this manuscript. "The funders had no role in study design, data collection and analysis, decision to publish, or preparation of the manuscript."

The causes of admission and death of manatees are defined, as now noted in the manuscript, by the Florida Fish and Wildlife Conservation Commission. In our opinion this reads well and is the vernacular that is used both within our community and with the public at large when communicating about manatees. We are not sure a table would be useful.

A detailed map showing all the rescue points would indeed be interesting but we believe too detailed for the purposes intended here. Work of this nature would be most appropriate as part of a thesis. The current manuscript is not delving into outcomes of the manatee rehabilitation; that is a follow-up manuscript so we think the descriptors are adequate. It is indeed an important aspect of the entire endeavor but again we are looking to focus our current efforts to the admission and see if there are lessons we can share.

Figures 5 and 6 do look similar but the point of each is distinctly different. Figure 5, with a revised caption, highlights the overall increase in manatee admissions over time in 5 year blocks. Figure 6 highlights the seasonality of admissions based on the admission categories.

We agree that some comparison to manatee rescues outside the USA would be appropriate. Brazil is really the only other country that does this with any frequency and that is the basis of the comparison I have added. It becomes problematic to compare facilities with the USA and within Florida as each facility is a private business, both non-profit and profit making operations. Sharing of medical data can be a challenge on an open basis that would include publication. One objective of this manuscript is to start to break down that barrier. With the completion of this manuscript, perhaps other facilities will then be open to compile their own information and data can be compared then. The fact that such a review as we are undertaking has *not* taken place yet really speaks volumes. This point is actually mentioned in the manuscript in lines 212 - 214.

The corrections have been made on lines 124-125 and thank you for detecting that discretion.

A paragraph has been added in the discussion regarding manatees rescues in Brazil, which is the next largest program involved with manatees. This discussion does provide a stark contrast to the situation we see in Florida and I do think this inclusion is a good improvement.

The last points from Reviewer 3 are exactly the questions we hope this manuscript raises and we are delighted to see this response. We would like to simply pose these questions outright in the manuscript with some thoughts and leave it unanswered as an invitation or encouragement for this work to continue.
